# How Many Bits Does it Take to Quantize Your Neural Network?

**DOI:** 10.1007/978-3-030-45237-7_5

**Published:** 2020-03-13

**Authors:** Mirco Giacobbe, Thomas A. Henzinger, Mathias Lechner

**Affiliations:** 8 Johannes Kepler University, Linz, Austria; 9 University of Birmingham, Birmingham, UK; 10 IST Austria, Klosterneuburg, Austria; 11 University of Oxford, Oxford, United Kingdom

## Abstract

Quantization converts neural networks into low-bit fixed-point computations which can be carried out by efficient integer-only hardware, and is standard practice for the deployment of neural networks on real-time embedded devices. However, like their real-numbered counterpart, quantized networks are not immune to malicious misclassification caused by adversarial attacks. We investigate how quantization affects a network’s robustness to adversarial attacks, which is a formal verification question. We show that neither robustness nor non-robustness are monotonic with changing the number of bits for the representation and, also, neither are preserved by quantization from a real-numbered network. For this reason, we introduce a verification method for quantized neural networks which, using SMT solving over bit-vectors, accounts for their exact, bit-precise semantics. We built a tool and analyzed the effect of quantization on a classifier for the MNIST dataset. We demonstrate that, compared to our method, existing methods for the analysis of real-numbered networks often derive false conclusions about their quantizations, both when determining robustness and when detecting attacks, and that existing methods for quantized networks often miss attacks. Furthermore, we applied our method beyond robustness, showing how the number of bits in quantization enlarges the gender bias of a predictor for students’ grades.

## 1 Introduction

Deep neural networks are powerful machine learning models, and are becoming increasingly popular in software development. Since recent years, they have pervaded our lives: think about the language recognition system of a voice assistant, the computer vision employed in face recognition or self driving, not to talk about many decision-making tasks that are hidden under the hood. However, this also subjects them to the resource limits that real-time embedded devices impose. Mainly, the requirements are low energy consumption, as they often run on batteries, and low latency, both to maintain user engagement and to effectively interact with the physical world. This translates into specializing our computation by reducing the memory footprint and instruction set, to minimize cache misses and avoid costly hardware operations. For this purpose, quantization compresses neural networks, which are traditionally run over 32-bit floating-point arithmetic, into computations that require bit-wise and integer-only arithmetic over small words, e.g., 8 bits. Quantization is the standard technique for the deployment of neural networks on mobile and embedded devices, and is implemented in TensorFlow Lite [13]. In this work, we investigate the robustness of quantized networks to adversarial attacks and, more generally, formal verification questions for quantized neural networks.

Adversarial attacks are a well-known vulnerability of neural networks [24]. For instance, a self-driving car can be tricked into confusing a stop sign with a speed limit sign [9], or a home automation system can be commanded to deactivate the security camera by a voice reciting poetry [22]. The attack is carried out by superposing the innocuous input with a crafted perturbation that is imperceptible to humans. Formally, the attack lies within the neighborhood of a known-to-be-innocuous input, according to some notion of distance. The fraction of samples (from a large set of test inputs) that do not admit attacks determines the robustness of the network. We ask ourselves how quantization affects a network's robustness or, dually, how many bits it takes to ensure robustness above some specific threshold. This amounts to proving that, for a set of given quantizations and inputs, there does not exists an attack, which is a formal verification question.

The formal verification of neural networks has been addressed either by overapproximating—as happens in abstract interpretation—the space of outputs given a space of attacks, or by searching—as it happens in SMT-solving—for a variable assignment that witnesses an attack. The first category include methods that relax the neural networks into computations over interval arithmetic [20], treat them as hybrid automata [27], or abstract them directly by using zonotopes, polyhedra [10], or tailored abstract domains [23]. Overapproximation-based methods are typically fast, but incomplete: they prove robustness but do not produce attacks. On the other hand, methods based on local gradient descent have turned out to be effective in producing attacks in many cases [16], but sacrifice formal completeness. Indeed, the search for adversarial attack is NP-complete even for the simplest (i.e., ReLU) networks [14], which motivates the rise of methods based on *Satisfiability Modulo Theory* (SMT) and *Mixed Integer Linear Programming* (MILP). SMT-solvers have been shown not to scale beyond toy examples (20 hidden neurons) on monolithic encodings [21], but today's specialized techniques can handle real-life benchmarks such as, neural networks for the MNIST dataset. Specialized tools include DLV [12], which subdivides the problem into smaller SMT instances, and Planet [8], which combines different SAT and LP relaxations. Reluplex takes a step further augmenting LP-solving with a custom calculus for ReLU networks [14]. At the other end of the spectrum, a recent MILP formulation turned out effective using off-the-shelf solvers [25]. Moreover, it formed the basis for Sherlock [7], which couples local search and MILP, and for a specialized branch and bound algorithm [4].

All techniques mentioned above do not reason about the machine-precise semantics of the networks, neither over floating- nor over fixed-point arithmetic, but reason about a real-number relaxation. Unfortunately, adversarial attacks computed over the reals are not necessarily attacks on execution architectures, in particular, for quantized networks implementations. We show, for the first time, that attacks and, more generally, robustness and vulnerability to attacks do not always transfer between real and quantized networks, and also do not always transfer monotonically with the number of bits across quantized networks. Verifying the real-valued relaxation of a network may lead scenarios where

(i) specifications are fulfilled by the real-valued network but not for its quantized implementation (false negative),
(ii) specifications are violated by the real-valued network but fulfilled by its quantized representation (false negatives), or
(iii) counterexamples witnessing that the real-valued network violated the specification, but do not witness a violation for the quantized network (invalid counterexamples/attacks).

More generally, we show that all three phenomena can occur non-monotonically with the precision in the numerical representation. In other words, it may occur that a quantized network fulfills a specification while both a higher and a lower bits quantization violate it, or that the first violates it and both the higher and lower bits quantizations fulfill it; moreover, specific counterexamples may not transfer monotonically across quantizations.

The verification of real-numbered neural networks using the available methods is inadequate for the analysis of their quantized implementations, and the analysis of quantized neural networks needs techniques that account for their bit-precise semantics. Recently, a similar problem has been addressed for binarized neural networks, through SAT-solving [18]. Binarized networks represent the special case of 1-bit quantizations. For many-bit quantizations, a method based on gradient descent has been introduced recently [28]. While efficient (and sound), this method is incomplete and may produce false negatives.

We introduce, for the first time, a complete method for the formal verification of quantized neural networks. Our method accounts for the bit-precise semantics of quantized networks by leveraging the first-order theory of bit vectors without quantifiers (QF_BV), to exactly encode hardware operations such as 2'complementation, bit-shift, integer arithmetic with overflow. On the technical side, we present a novel encoding which balances the layout of long sequences of hardware multiply-add operations occurring in quantized neural networks. As a result, we obtain a encoding into a first-order logic formula which, in contrast to a standard unbalanced linear encoding, makes the verification of quantized networks practical and amenable to modern bit-precise SMT-solving. We built a tool using Boolector [19], evaluated the performance of our encoding, compared its effectiveness against real-numbered verification and gradient descent for quantized networks, and finally assessed the effect of quantization for different networks and verification questions.

We measured the robustness to attacks of a neural classifier involving 890 neurons and trained on the MNIST dataset (handwritten digits), for quantizations between 6 and 10 bits. First, we demonstrated that Boolector, off-the-shelf and using our balanced SMT encoding, can compute every attack within 16

hours, with a median time of 3h 41m, while timed-out on all instances beyond 6 bits using a standard linear encoding. Second, we experimentally confirmed that both Reluplex and gradient descent for quantized networks can produce false conclusions about quantized networks; in particular, spurious results occurred consistently more frequently as the number of bits in quantization decreases. Finally, we discovered that, to achieve an acceptable level of robustness, it takes a higher bit quantization than is assessed by standard accuracy measures.

Lastly, we applied our method beyond the property of robustness. We also evaluate the effect of quantization upon the gender bias emerging from quantized predictors for students' performance in mathematics exams. More precisely, we computed the maximum predictable grade gap between any two students with identical features except for gender. The experiment showed that a substantial gap existed and was proportionally enlarged by quantization: the lower the number bits the larger the gap.

We summarize our contribution in five points. First, we show that the robustness of quantized neural networks is non-monotonic in the number of bits and is non-transferable from the robustness of their real-numbered counterparts. Second, we introduce the first complete method for the verification of quantized neural networks. Third, we demonstrate that our encoding, in contrast to standard encodings, enabled the state-of-the-art SMT-solver Boolector to verify quantized networks with hundreds of neurons. Fourth, we also show that existing methods determine both robustness and vulnerability of quantized networks less accurately than our bit-precise approach, in particular for low-bit quantizations. Fifth, we illustrate how quantization affects the robustness of neural networks, not only with respect to adversarial attacks, but also with respect to other verification questions, specifically fairness in machine learning.

## 2 Quantization of Feed-forward Networks

A feed-forward neural network consists of a finite set of *neurons* $x_1, \ldots, x_k$ partitioned into a sequence of layers: an *input layer* with $n$ neurons, followed by one or many *hidden layers*, finally followed by an *output layer* with $m$ neurons. Every pair of neurons $x_j$ and $x_i$ in respectively subsequent layers is associated with a *weight* coefficient $w_{ij} \in \mathbb{R}$; if the layer of $x_j$ is not subsequent to that of $x_i$, then we assume $w_{ij} = 0$. Every hidden or output neuron $x_i$ is associated with a *bias* coefficient $b_i \in \mathbb{R}$. The real-valued semantics of the neural network gives to each neuron a real value: upon a valuation for the neurons in the input layer, every other neuron $x_i$ assumes its value according to the update rule

$$x_i = \text{ReLU-}N(b_i + \sum_{j=1}^{k} w_{ij} x_j), \tag{1}$$

where ReLU-$N \colon \mathbb{R} \to \mathbb{R}$ is the *activation function.* Altogether, the neural network implements a function $f \colon \mathbb{R}^n \to \mathbb{R}^m$ whose result corresponds to the valuation for the neurons in the output layer.

The activation function governs the firing logic of the neurons, layer by layer, by introducing non-linearity in the system. Among the most popular activation functions are purely non-linear functions, such as the tangent hyperbolic and the sigmoidal function, and piece-wise linear functions, better known as *Rectified Linear Units* (ReLU) [17]. ReLU consists of the function that takes the positive part of its argument, i.e., $\text{ReLU}(x) = \max\{x, 0\}$. We consider the variant of ReLU that imposes a cap value $N$, known as ReLU-$N$ [15]. Precisely

$$\text{ReLU-}N(x) = \min\{\max\{x, 0\}, N\}, \tag{2}$$

which can be alternatively seen as a concatenation of two ReLU functions (see Eq. 10). As a consequence, all neural networks we treat are full-fledged ReLU networks; their real-valued versions are amenable to state-of-the-art verification tools including Reluplex, but neither account for the exact floating- nor fixed-point execution models.

Quantizing consists of converting a neural network over real numbers, which is normally deployed on floating-point architectures, into a neural network over integers, whose semantics corresponds to a computation over fixed-point arithmetic [13]. Specifically, fixed-point arithmetic can be carried out by integer-only architectures and possibly over small words, e.g., 8 bits. All numbers are represented in 2's complement over $B$ bits words and $F$ bits are reserved to the fractional part: we call the result a *$B$-bits quantization in QF arithmetic.* More concretely, the conversion follows from the rounding of weight and bias coefficients to the $F$-th digit, namely $\bar{b}_i = \text{rnd}(2^F b_i)$ and $\bar{w}_{ij} = \text{rnd}(2^F w_{ij})$ where $\text{rnd}(\cdot)$ stands for any rounding to an integer. Then, the fundamental relation between a quantized value $\bar{a}$ and its real counterpart $a$ is

$$a \approx 2^{-F}\bar{a}. \tag{3}$$

Consequently, the semantics of a quantized neural network corresponds to the update rule in Eq. 1 after substituting of $x$, $w$, and $b$ with the respective approximants $2^{-F}\bar{x}$, $2^{-F}\bar{w}$, and $2^{-F}\bar{b}$. Namely, the semantics amounts to

$$\bar{x}_i = \text{ReLU-}(2^F N)(\bar{b}_i + \text{int}(2^{-F} \sum_{j=1}^{k} \bar{w}_{ij}\bar{x}_j)), \tag{4}$$

where $\text{int}(\cdot)$ truncates the fractional part of its argument or, in other words, rounds towards zero. In summary, the update rule for the quantized semantics consists of four parts. The first part, i.e., the linear combination $\sum_{j=1}^{k} \bar{w}_{ij}\bar{x}_j$, propagates all neurons values from the previous layer, obtaining a value with possibly $2B$ fractional bits. The second scales the result by $2^{-F}$ truncating the fractional part by, in practice, applying an arithmetic shift to the right of $F$ bits. Finally, the third applies the bias $\bar{b}$ and the fourth clamps the result between 0 and $2^F N$. As a result, a quantize neural network realizes a function $f\colon \mathbb{Z}^n \to \mathbb{Z}^m$, which exactly represents the concrete (integer-only) hardware execution.

We assume all intermediate values, e.g., of the linear combination, to be fully representable as, coherently with the common execution platforms [13], we always allocate enough bits for under and overflow not to happen. Hence, any loss of precision from the respective real-numbered network happens exclusively, at each layer, as a consequence of rounding the result of the linear combination to $F$ fractional bits. Notably, rounding causes the robustness to adversarial attacks of quantized networks with different quantization levels to be independent of one another, and independent of their real counterpart.

## 3 Robustness is Non-monotonic in the Number of Bits

A neural classifier is a neural network that maps a $n$-dimensional input to one out of $m$ classes, each of which is identified by the output neuron with the largest value, i.e., for the output values $z_1, \ldots, z_m$, the choice is given by

$$\text{class}(z_1, \ldots, z_m) = \arg\max_i z_i. \tag{5}$$

For example, a classifier for handwritten digits takes in input the pixels of an image and returns 10 outputs $z_0, \ldots, z_9$, where the largest indicates the digit the image represents. An adversarial attack is a perturbation for a sample input

$$\text{original} + \text{perturbation} = \text{attack}$$

that, according to some notion of closeness, is indistinguishable from the original, but tricks the classifier into inferring an incorrect class. The attack in Fig. 1 is

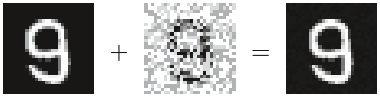

**Fig. 1:** Adversarial attack.

indistinguishable from the original by the human eye, but induces our classifier to assign the largest value to $z_3$, rather than $z_9$, misclassifying the digit as a 3. For this example, misclassification happens consistently, both on the real-numbered and on the respective 8-bits quantized network in Q4 arithmetic. Unfortunately, attacks do not necessarily transfer between real and quantized networks and neither between quantized networks for different precision. More generally, attacks and, dually, robustness to attacks are non-monotonic with the number of bits.

We give a prototypical example for the non-monotonicity of quantized networks in Fig. 2. The network consists of one input, 4 hidden, and 2 output neurons, respectively from left to right. Weights and bias coefficients, which are annotated on the edges, are all fully representable in Q1. For the neurons in the top row we show, respectively from top to bottom, the valuations obtained using a Q3, Q2, and Q1 quantization of the network (following Eq. 4); precisely, we

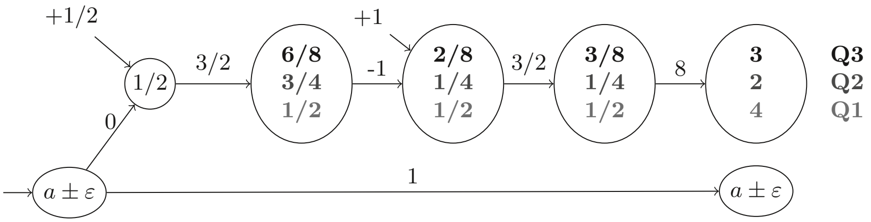

**Fig. 2:** Neural network with non-monotonic robustness w.r.t. its Q1, Q2, and Q3 quantizations.

show their fractional counterpart $\bar{x}/2^F$. We evaluate all quantizations and obtain that the valuations for the top output neuron are non-monotonic with the number of fractional bits; in fact, the Q1 dominates the Q3 which dominates the Q2 output. Coincidentally, the valuations for the Q3 quantization correspond to the valuations with real-number precision (i.e., never undergo truncation), indicating that also real and quantized networks are similarly incomparable. Notably, all phenomena occur both for quantized networks with rounding towards zero (as we show in the example), and with rounding to the nearest, which is naturally non-monotonic (e.g., 5/16 rounds to 1/2, 1/4, and 3/8 with, resp., Q1, Q2, and Q3).

Non-monotonicity of the output causes non-monotonicity of robustness, as we can put the decision boundary of the classifier so as to put Q2 into a different class than Q1 and Q3. Suppose the original sample is 3/2 and its class is associated with the output neuron on the top, and suppose attacks can only lay in the neighboring interval $3/2 \pm 1$. In this case, we obtain that the Q2 network admits an attack, because the bottom output neuron can take 5/2, that is larger than 2. On the other hand, the bottom output can never exceed 3/8 and 1/2, hence Q1 and Q3 are robust. Dually, also non-robustness is non-monotonic as, for the sample 9/2 whose class corresponds to the bottom neuron, for the interval 9/2 $\pm$ 2, Q2 is robust while both Q3 and Q1 are vulnerable. Notably, the specific attacks of Q3 and Q1 also do not always coincide as, for instance, 7/2.

Robustness and non-robustness are non-monotonic in the number of bits for quantized networks. As a consequence, verifying a high-bits quantization, or a real-valued network, may derive false conclusions about a target lower-bits quantization, in either direction. Specifically, for the question as for whether an attack exists, we may have both (i) false negatives, i.e., the verified network is robust but the target network admits an attack, and (ii) false positives, i.e., the verified network is vulnerable while the target network robust. In addition, we may also have (iii) true positives with invalid attacks, i.e., both are vulnerable but the found attack do not transfer to the target network. For these reasons we introduce a verification method quantized neural network that accounts for their bit-precise semantics.

## 4 Verification of Quantized Networks using Bit-precise SMT-solving

Bit-precise SMT-solving comprises various technologies for deciding the satisfiability of first-order logic formulae, whose variables are interpreted as bit-vectors of fixed size. In particular, it produces satisfying assignments (if any exist) for formulae that include bitwise and arithmetic operators, whose semantics corresponds to that of hardware architectures. For instance, we can encode bit-shifts, 2's complementation, multiplication and addition with overflow, signed and unsigned comparisons. More precisely, this is the quantifier-free first-order theory of bit-vectors (i.e., QF_BV), which we employ to produce a monolithic encoding of the verification problem for quantized neural networks.

A verification problem for the neural networks $f_1, \dots, f_K$ consists of checking the validity of a statement of the form

$$\varphi(\boldsymbol{y}_1, \dots, \boldsymbol{y}_K) \implies \psi(f_1(\boldsymbol{y}_1), \dots, f_K(\boldsymbol{y}_K)), \tag{6}$$

where $\varphi$ is a predicate over the inputs and $\psi$ over the outputs of all networks; in other words, it consists of checking an input–output relation, which generalizes various verification questions, including robustness to adversarial attacks and fairness in machine learning, which we treat in Sec. 5. For the purpose of SMT solving, we encode the verification problem in Eq. 6, which is a validity question, by its dual satisfiability question

$$\varphi(\boldsymbol{y}_1, \dots, \boldsymbol{y}_K) \wedge \bigwedge_{i=1}^{K} f_i(\boldsymbol{y}_i) = \boldsymbol{z}_i \ \wedge \ \neg\psi(\boldsymbol{z}_1, \dots, \boldsymbol{z}_K), \tag{7}$$

whose satisfying assignments constitute counterexamples for the contract. The formula consists of three conjuncts: the rightmost constraints the input within the assumption, the leftmost forces the output to violate the guarantee, while the one in the middle relates inputs and outputs by the semantics of the neural networks.

The semantics of the network consists of the bit-level translation of the update rule in Eq. 4 over all neurons, which we encode in the formula

$$\bigwedge_{i=1}^{k} x_i = \text{ReLU-}(2^F N)(x_i') \wedge \ x_i' = \bar{b}_i + \texttt{ashr}(x_i'', F) \wedge \ x_i'' = \sum_{j=1}^{k} \bar{w}_{ij} x_j. \tag{8}$$

Each conjunct in the formula employs three variables $x$, $x'$, and $x''$ and is made of three, respective, parts. The first part accounts for the operation of clamping between 0 and $2^F N$, whose semantics is given by the formula ReLU-$M(x) = \texttt{ite}(\texttt{sign}(x), 0, \texttt{ite}(x \geq M, M, x))$. Then, the second part accounts for the operations of scaling and biasing. In particular, it encodes the operation of rounding by truncation scaling, i.e., $\text{int}(2^{-F}x)$, as an arithmetic shift to the right. Finally, the last part accounts for the propagation of values from the previous layer, which, despite the obvious optimization of pruning away all monomials

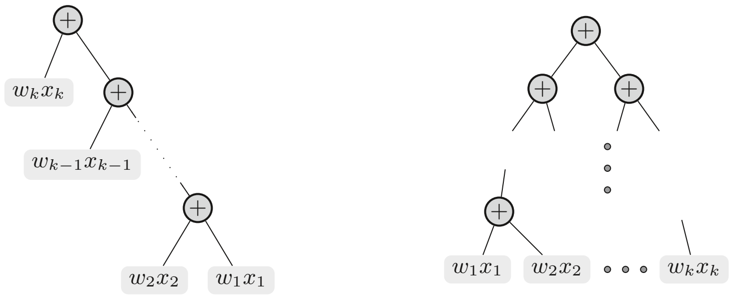

Linear layout
(a)

Balanced layout
(b)

**Fig. 3:** Abstract syntax trees for alternative encodings of a long linear combination of the form $\sum_{i=1}^{k} w_i x_i$.

with null coefficient, often consists of long linear combinations, whose exact semantic amounts to a sequence of multiply-add operations over an accumulator; particularly, encoding it requires care in choosing variables size and association layout.

The size of the bit-vector variables determines whether overflows can occur. In particular, since every monomial $w_{ij}x_j$ consists of the multiplication of two $B$-bits variables, its result requires $2B$ bits in the worst case; since summation increases the value linearly, its result requires a logarithmic amount of extra bits in the number of summands (regardless of the layout). Provided that, we avoid overflow by using variables of $2B + \log k$ bits, where $k$ is the number of summands.

The association layout is not unique and, more precisely, varies with the order of construction of the long summation. For instance, associating from left to right produces a linear layout, as in Fig. 3a. Long linear combonations occurring in quantized neural networks are implemented as sequences of multiply-add operations over a single accumulator; this naturally induces a linear encoding. Instead, for the purpose formal verification, we propose a novel encoding which re-associates the linear combination by recursively splitting the sum into equal parts, producing a *balanced layout* as in Fig. 3b. While linear and balanced layouts are semantically equivalent, we have observed that, in practice, the second impacted positively the performance of the SMT-solver as we discuss in Sec. 5, where we also compare against other methods and investigate different verification questions.

## 5 Experimental Results

We set up an experimental evaluation benchmark based on the MNIST dataset to answer the following three questions. First, how does our balanced encoding scheme impact the runtime of different SMT solvers compared to a standard linear encoding? Then, how often can robustness properties, that are proven for the real-valued network, transferred to the quantized network and vice versa? Finally, how often do gradient based attacking procedures miss attacks for quantized networks?

The MNIST dataset is a well-studied computer vision benchmark, which consists of 70,000 handwritten digits represented by 28-by-28 pixel images with a single 8-bit grayscale channel. Each sample belongs to exactly one category $\{0, 1, \ldots 9\}$, which a machine learning model must predict from the raw pixel values. The MNIST set is split into 60,000 training and 10,000 test samples.

We trained a neural network classifier on MNIST, following a *post-training quantization* scheme [13]. First, we trained, using TensorFlow with floating-point precision, a network composed of 784 inputs, 2 hidden layers of size 64, 32 with ReLU-7 activation function and 10 outputs, for a total of 890 neurons. The classifier yielded a *standard accuracy*, i.e., the ratio of samples that are correctly classified out of all samples in the testing set, of 94.7% on the floating-point architecture. Afterward, we quantized the network with various bit sizes, with the exception of imposing the input layer to be always quantized in 8 bits, i.e., the original precision of the samples. The quantized networks required at least Q3 with 7 total bits to obtain an accuracy above 90% and Q5 with 10 bits to reach 94%. For this reason, we focused our study on the quantizations from 6 and the 10 bits in, respectively, Q2 to Q6 arithmetic.

Robust accuracy or, more simply, *robustness* measure the ratio of robust samples: for the distance $\varepsilon > 0$, a sample $a$ is robust when, for all its perturbations $y$ within that distance, the classifier $\text{class} \circ f$ chooses the original class $c = \text{class} \circ f(a)$. In other words, $a$ is robust if, for all $\boldsymbol{y}$

$$|a - \boldsymbol{y}|_\infty \leq \varepsilon \implies c = \text{class} \circ f(\boldsymbol{y}), \tag{9}$$

where, in particular, the right-hand side can be encoded as $\bigwedge_{j=1}^{m} z_j \leq z_c$, for $\boldsymbol{z} = f(\boldsymbol{y})$. Robustness is a validity question as in Eq. 6 and any witness for the dual satisfiability question constitutes an adversarial attack. We checked the robustness of our selected networks over the first 300 test samples from the dataset with $\varepsilon = 1$ on the first 200 and $\varepsilon = 2$ on the next 100; in particular, we tested our encoding using the SMT-solver Boolector [19], Z3 [5], and CVC4 [3], off-the-shelf.

Our experiments serve two purposes. The first is evaluating the scalability and precision of our approach. As for scalability, we study how encoding layout, i.e., linear or balanced, and the number of bits affect the runtime of the SMT-solver. As for precision, we measured the gap between our method and both a formal verifier for real-numbered networks, i.e., Reluplex [14], and the IFGSM algorithm [28], with respect to the accuracy of identifying robust and vulnerable samples. The second purpose of our experiments is evaluating the effect of quantization on the robustness to attacks of our MNIST classifier and, with an additional experiment, measuring the effect of quantization over the gender fairness of a student grades predictor, also demonstrating the expressiveness of our method beyond adversarial attacks.

As we only compared the verification outcomes, any complete verifier for real-numbered networks would lead to the same results as those obtained with Reluplex. Note that these tools verify the real-numbered abstraction of the network using some form of linear real arithmetic reasoning. Consequently, rounding errors introduced by the floating-point implementation of both, the network and the verifier, are not taken into account.

### 5.1 Scalability and performance

We evaluated whether our balanced encoding strategy, compared to a standard linear encoding, can improve the scalability of contemporary SMT solvers for quantifier-free bit-vectors (QF_BV) to check specifications of quantized neural networks. We ran all our experiments on an Intel Xeon W-2175 CPU, with 64GB memory, 128GB swap file, and 16 hours of time budget per problem instance. We encoded each instance using the two variants, the standard linear and our balanced layout. We scheduled 14 solver instances in parallel, i.e., the number of physical processor cores available on our machine. While Z3, CVC4 and Yices2

| SMT-solver | Encoding | 6-bit | 7-bit | 8-bit | 9-bit | 10-bit |
|---|---|---|---|---|---|---|
| Boolector [19] | Linear (standard) | 3h 25m | oot | oot | oot | oot |
| | Balanced (ours) | 18m | 1h 29m | 3h 41m | 5h 34m | 8h 58m |
| Z3 [5] | Linear (standard) | oot | - | - | - | - |
| | Balanced (ours) | oot | - | - | - | - |
| CVC4 [3] | Linear (standard) | oom | - | - | - | - |
| | Balanced (ours) | oom | - | - | - | - |
| Yices2 [6] | Linear (standard) | oot | - | - | - | - |
| | Balanced (ours) | oot | - | - | - | - |

**Table 1:** Median runtimes for bit-exact robustness checks. The term oot refers to timeouts, and oom refers to out-of-memory errors. Due to the poor performance of Z3, CVC4, and Yices2 on our smallest 6-bit network, we abstained from running experiments involving more than 6 bits, i.e., entries marked by a dash (-).

timed out or ran out of memory on the 6-bit network, Boolector could check the instances of our smallest network within the given time budget, independently of the employed encoding scheme. Our results align with the SMT-solver performances reported by the SMT-COMP 2019 competition in the QF_BV division [11]. Consequently, we will focus our discussion on the results obtained with Boolector.

With linear layout Boolector timed-out on all instances but the smallest networks (6 bits), while with the balanced layout it checked all instances with an overall median runtime of 3h 41m and, as shown in Tab. 1, roughly doubling at every bits increase, as also confirmed by the histogram in Fig. 4.

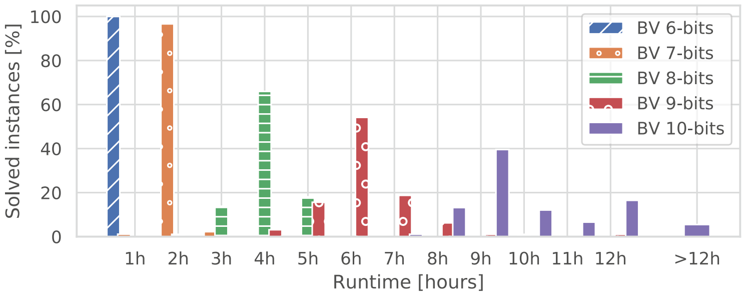

**Fig. 4:** Runtimes for bit-exact adversarial robustness checks of a classifier trained on the MNIST dataset using Boolector and our balanced SMT encodings. Runtime roughly doubles with each additional bit used for the quantization.

Our results demonstrate that our balanced association layout improves the performance of the SMT-solver, enabling it to scale to networks beyond 6 bits. Conversely, a standard linear encoding turned out to be ineffective on all tested SMT solvers. Besides, our method tackled networks with 890 neurons which, while small compared to state-of-the-art image classification models, already pose challenging benchmarks for the formal verification task. In the real-numbered world, for instance, off-the-shelf solvers could initially tackle up to 20 neurons [20], and modern techniques, while faster, are often evaluated on networks below 1000 neurons [14,4].

Additionally, we pushed our method to its limits, refining our MNIST network to a four-layers deep Convolutional network (2 Conv + 2 Fully-connected layers) with a total of 2238 neurons, which achieved a test accuracy of 98.56%. While for the 6-bits quantization we proved robustness for 99% of the tested samples within a median runtime of 3h 39min, for 7-bits and above all instances timed-out. Notably, Reluplex also failed on the real-numbered version, reporting numerical instability.

### 5.2 Comparison to other methods

Looking at existing methods for verification, one has two options to verify quantized neural networks: verifying the real-valued network and hoping the functional property is preserved when quantizing the network, or relying on incomplete methods and hoping no counterexample is missed. A question that emerges is how accurate are these two approaches for verifying robustness of a quantized network? To answer this question, we used Reluplex [14] to prove the robustness of the real-valued network. Additionally, we compared to the Iterative Fast Gradient Sign Method (IFGSM), which has recently been proposed to generate $\ell_\infty$-bounded adversarial attacks for quantized networks [28]; notably, IFGSM is incomplete in the sense that it may miss attacks. We then compared these two verification outcomes to the ground-truth obtained by our approach.

In our study, we employ the following notation. We use the term "false negative" (i) to describe cases in which the quantized network can be attacked, while no attack exists that fools the real-number network. Conversely, the term "false positive" (ii) describes the cases in which a real-number attack exists while the quantized network is robust. Furthermore, we use the term "invalid attack" (iii) to specify attacks produced for the real-valued network that fools the real-valued network but not the quantized network.

Regarding the real-numbered encoding, Reluplex accepts only pure ReLU networks. For this reason, we translate our ReLU-$N$ networks into functionally equivalent ReLU networks, by translating each layer with

$$\text{ReLU-}N(W \cdot \boldsymbol{x} + \boldsymbol{b}) = \text{ReLU}\Big( - I \cdot \text{ReLU}(-W \cdot \boldsymbol{x} - \boldsymbol{b} + N)\Big). \tag{10}$$

Out of the 300 samples, at least one method timed out on 56 samples, leaving us with 244 samples whose results were computed over all networks. Tab. 2 depicts how frequently the robustness property could be transferred from the real-valued network to the quantized networks. Not surprisingly, we observed the trend that when increasing the precision of the network, the error between the quantized model and the real-valued model decreases. However, even for the 10-bit model, in 0.8% of the tested samples, verifying the real-valued model leads to a wrong conclusion about the robustness of the quantized network. Moreover, our results show the existence of samples where the 10-bit network is robustness while the real-valued is attackable and vice versa. The invalid attacks illustrate that the higher the precision of the quantization, the more targeted attacks need to be. For instance, while 94% of attacks generated for the real-valued network represented valid attacks on the 7-bit model, this percentage decrease to 80% for the 10-bit network.

| Bits | True negatives | False negatives (i) | False positives (ii) | True positives | Invalid attacks (iii) |
|---|---|---|---|---|---|
| 6 | 66.4% | 25.0% | 3.3% | 5.3% | 8% |
| 7 | 84.8% | 6.6% | 1.6% | 7.0% | 6% |
| 8 | 88.5% | 2.9% | 0.4% | 8.2% | 10% |
| 9 | 91.0% | 0.4% | 0.4% | 8.2% | 20% |
| 10 | 91.0% | 0.4% | 0.4% | 8.2% | 20% |

**Table 2:** Transferability of vulnerability from the verification outcome of the real-valued network to the verification outcome of the quantized model. While vulnerability is transferable between the real-valued and the higher precision networks, (9 and 10-bits), in most of the tested cases, this discrepancy significantly increases when compressing the networks with fewer bits, i.e. see columns (i) and (ii).

Next, we compared how well incomplete methods are suited to reason about the robustness of quantized neural networks. We employed IFGSM to attack the 244 test samples for which we obtained the ground-truth robustness and measure how often IFGSM is correct about assessing the robustness of the network. For the sake of completeness, we perform the same analysis for the real-valued network.

| Bits | True negatives | False negatives (i) | False positives (ii) | True positives |
|---|---|---|---|---|
| 6 | 69.7% | 1.2 % | - | 30.3% |
| 7 | 86.5% | 1.6 % | - | 13.5% |
| 8 | 88.9% | 0.8 % | - | 11.1% |
| 9 | 91.4% | 0.8 % | - | 8.6 % |
| 10 | 91.4% | 0 % | - | 8.6 % |
| $\mathbb{R}$ | 91.4% | 0 % | - | 8.6 % |

**Table 3:** Transferability of incomplete robustness verification (IFGSM [28]) to ground-truth robustness (ours) for quantized networks. While for the real-valued and 10-bit networks our gradient based incomplete verification did not miss any possible attack, a non-trivial number of vulnerabilities were missed by IFGSM for the low-bit networks. The row indicted by $\mathbb{R}$ compares IFGSM attacking the floating-point implementation to the grouth-truth obtained, using Reluplex, by verifying the real-valued relaxation of the network.

Our results in Tab. 3 present the trend that with higher precision, e.g., 10-bits or reals, incomplete methods provide a stable estimate about the robustness of the network, i.e., IFGSM was able to find attacks for all non-robust samples. However, for lower precision levels, IFGSM missed a substantial amount of attacks, i.e., for the 7-bit network, IFGSM could not find a valid attack for 10% of the non-robust samples.

### 5.3 The effect of quantization on robustness

In Tab. 3 we show how standard accuracy and robust accuracy degrade on our MNIST classifier when increasing the compression level. The data indicates a constant discrepancy between standard accuracy and robustness; for real numbered networks, a similar fact was already known in the literature [26]: we empirically confirm that observation for our quantized networks, whose discrepancy fluctuated between 3 and 4% across all precision levels. Besides, while an acceptable, larger than 90%, standard accuracy was achieved at 7 bits, an equally acceptable robustness was achieved at 9 bits.

One relationship not shown in Tab. 3 is that these 4% of non-robust samples are not equal for across quantization levels. For instance, we observed samples

| Precision | 6 | 7 | 8 | 9 | 10 | $\mathbb{R}$ |
|---|---|---|---|---|---|---|
| Standard | 73.4% | 91.8% | 92.2% | 94.3% | 95.5% | 94.7% |
| Robust | 69.7% | 86.5% | 88.9% | 91.4% | 91.4% | 91.4% |

**Table 4:** Accuracy of the MNIST classifiers on the 244 test samples for which all quantization levels could be check within the given time budget. The column indicated by $\mathbb{R}$ compares the accuracy of the floating-point implementation to the robust accuracy of the real-valued relaxation of the network.

that are robust for 7-bit network but attackable when quantizing with 9- and 10-bits. Conversely, there are attacks for the 7-bit networks that are robust samples in the 8-bit network.

### 5.4 Network specifications beyond robustness

Concerns have been raised that decisions of an ML system could discriminate towards certain groups due to a bias in the training data [2]. A vital issue in quantifying fairness is that neural networks are black-boxes, which makes it hard to explain how each input contributes to a particular decision.

We trained a network on a publicly available dataset consisting of 1000 students' personal information and academic test scores [1]. The personal features include gender, parental level of education, lunch plans, and whether the student took a preparation course for the test, all of which are discrete variables. We train a predictor for students' math scores, which is a discrete variable between 0 and 100. Notably, the dataset contains a potential source for gender bias: the mean math score among females is 63.63, while it is 68.73 among males.

The network we trained is composed of 2 hidden layers with 64 and 32 units, respectively. We use a 7-bit quantization-aware training scheme, achieving a 4.14% mean absolute error, i.e., the difference between predicted and actual math scores on the test set.

The network is *fair* if the gender of a person influences the predicted math score by at most the bias $\beta$. In other words, checking fairness amounts to verifying that

$$\bigwedge_{i \neq \text{gender}} s_i = t_i \wedge s_{\text{gender}} \neq t_{\text{gender}} \implies |f(\boldsymbol{s}) - f(\boldsymbol{t})| \leq \beta, \tag{11}$$

is valid over the variables $\boldsymbol{s}$ and $\boldsymbol{t}$, which respectively model two students for which gender differs but all other features are identical—we call them twin students. When we encode the dual formula, we encode two copies of the semantics of the same network: to one copy we give one student $\boldsymbol{s}$ and take the respective grade $g$, to the other we give its twin $\boldsymbol{t}$ and take grade $h$; precisely, we check for the unsatisfiability the negation of formula in Eq. 11. Then, we compute a tight upper bound for the bias, that is the maximum possible change in predicted score for any two twins. To compute the tightest bias, we progressively increase $\beta$ until our encoded formula becomes unsatisfiable.

We measure mean test error and gender bias of the 6- to the 10-bits quantization of the networks. We show the results in Tab. 5. The test error was stable

| Quantization level | Mean test error | Tightest bias upper bound |
|---|---|---|
| 6 bits | 4.46 | 22 |
| 7 bits | 4.14 | 17 |
| 8 bits | 4.37 | 16 |
| 9 bits | 4.38 | 15 |
| 10 bits | 4.59 | 15 |

**Table 5:** Results for the formal analysis of the gender bias of a students' grade predictor. The maximum gender bias of the network monotonically decreases with increasing precision.

between 4.1 and 4.6% among all quantizations, showing that the change in precision did not affect the quality of the network in a way that was perceivable by standard measures. However, our formal analysis confirmed a gender bias in the network, producing twins with a 15 to 21 difference in predicted math score. Surprisingly, the bias monotonically increased as the precision level in quantization lowered, indicating to us that quantization plays a role in determining the bias.

## 6 Conclusion

We introduced the first complete method for the verification of quantized neural networks which, by SMT solving over bit-vectors, accounts for their bit-precise semantics. We demonstrated, both theoretically and experimentally, that bit-precise reasoning is necessary to accurately ensure the robustness to adversarial attacks of a quantized network. We showed that robustness and non-robustness are non-monotonic in the number of bits for the numerical representation and that, consequently, the analysis of high-bits or real-numbered networks may derive false conclusions about their lower-bits quantizations. Experimentally, we confirmed that real-valued solvers produce many spurious results, especially on low-bit quantizations, and that also gradient descent may miss attacks. Additionally, we showed that quantization indeed affects not only robustness, but also other properties of neural networks, such as fairness. We also demonstrated that, using our balanced encoding, off-the-shelf SMT-solving can analyze networks with hundreds of neurons which, despite hitting the limits of current solvers, establishes an encouraging baseline for future research.

## Acknowledgments

An early version of this paper was put into the easychair repository as EasyChair Preprint no. 1000. This research was supported in part by the Austrian Science Fund (FWF) under grants S11402-N23(RiSE/SHiNE) and Z211-N23 (Wittgenstein Award), in part by the Aerospace Technology Institute (ATI), the Department for Business, Energy & Industrial Strategy (BEIS), and Innovate UK under the HICLASS project (113213).
